# P-515. Enhancing Sexual Wellness: A Novel Approach to Screening and Prevention in an Urban Health Center

**DOI:** 10.1093/ofid/ofae631.714

**Published:** 2025-01-29

**Authors:** Mitchell Caponi, Miriam Bonano, Eduardo Bayter, Kathryn Keneipp, Sandeep Bhat

**Affiliations:** The Family Health Centers at NYU Langone, Brooklyn, New York; Sunset Park Health Council, Bronx, New York; The Family Health Centers at NYU Langone, Brooklyn, New York; Family Health Centers at NYU Langone, Brooklyn, New York; NYU Langone, New York, New York

## Abstract

**Background:**

The Family Health Centers at NYU Langone (FHC) is a network of Federally Qualified Health Centers that serves a largely marginalized, diverse, and low-income patient population in Brooklyn, NY. In 2023, the FHC provided care for over 112,000 patients, with 52% being on Medicaid or Medicare and 28% being uninsured. We identified 44 HBSAG positive patients as well as 25 newly diagnosed HIV patients.

Patients as well as providers are often reluctant to have conversations around sexual health. Identifying risk factors for HIV, infectious hepatitis, and other infections is an area critical for providing excellent primary care to end the epidemic and curb Hep and STI rates.

**Figure:**
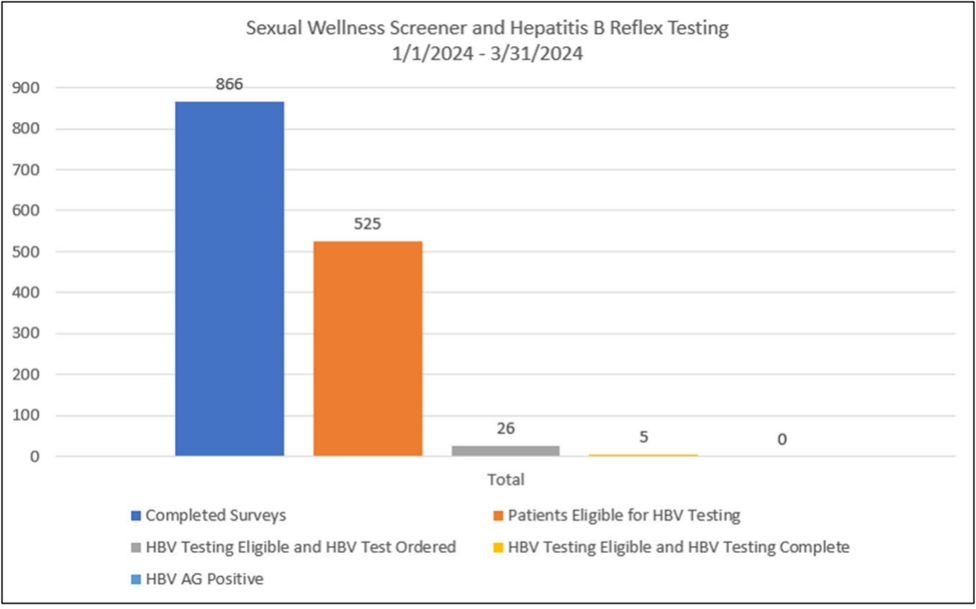

**Methods:**

The FHC had no standardized approach to collecting HIV and Hep B risk history being followed by providers. Our approach aimed to standardize risk screening processes while raising provider awareness of the significance of Hepatitis screening and HIV prevention.

A Sexual Wellness Screening Tool was developed and integrated into the EPIC My Chart system. The screening tool is answered by patients ahead of an initial/annual appointment through the patient portal and updated annually.

Patients with high-risk responses trigger an EPIC Best Practice Alert (BPA). The BPA recommends elements of care and prepopulates a Hepatitis B lab orders and/or referrals to the HIV prevention team. For provider buy-in and education, learning sessions were held across the network.

**Figure:**
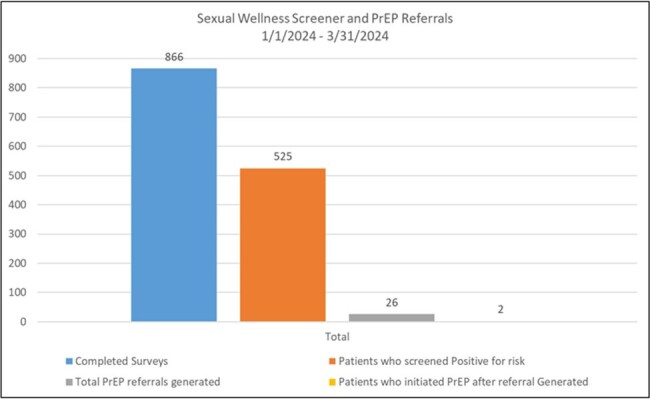

**Results:**

During Q1 of 2024, 866 patients completed the tool, 525 patients screening positive for risk factors. Of the 525 patients, 88% reported having sex without a condom in the past 12 months. 65% of patients had multiple risk factors. All patients at risk, were offered testing, however 26 HBV tests were actually ordered and 5 completed. For HIV prevention, 26 referrals were generated and contacted by Prevention Navigators, leading to 2 patients beginning PrEP.

Risk Responses

**Figure:**
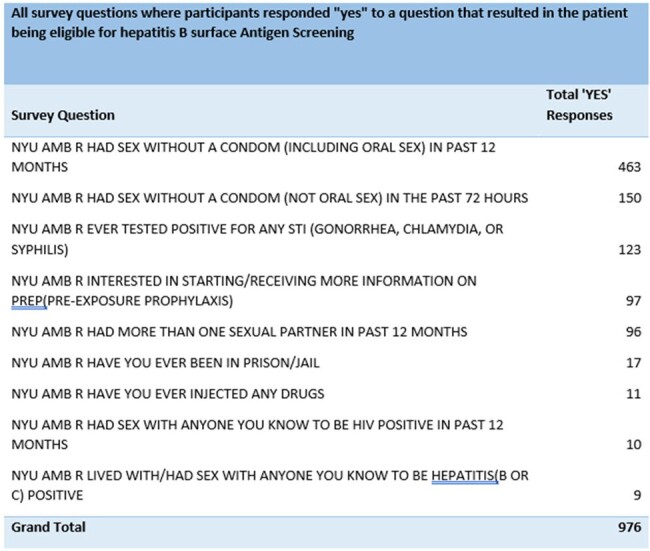

**Conclusion:**

Having patients fill out the tool ahead of appointments allows for a provider to review the tool with the patient and can help guide the conversations patients and providers have; maximizing the finite time providers have with patients and empowering patients to have their questions answered. Continued education and laboratory process refinement are necessary to streamline the work.

**Disclosures:**

**All Authors**: No reported disclosures

